# Evaluation of preoperative magnetic resonance imaging features and diagnostic effectiveness of grades II and III intracranial solitary fibroma

**DOI:** 10.1186/s40001-024-01959-5

**Published:** 2024-07-19

**Authors:** Yuncai Ran, Xiao Wang, Yong Zhang, Rui Chen, Chenchen Liu, Yunwei Ran, Weijian Wang, Xiaoyue Ma, Mengzhu Wang, Jingliang Cheng

**Affiliations:** 1 Magnetic Resonance Department, 1st Affiliated Hospital of Zhengzhou University, Zhengzhou, China; 2 Medical Research Center, The Third Affiliated Hospital of Zhengzhou University, Zhengzhou, China; 3 MR Research Collaboration, Siemens Healthineers Ltd., Beijing, China

**Keywords:** Intracranial solitary fibroma, Magnetic resonance imaging, Preoperative characterization, Apparent diffusion coefficient, T2 FLAIR

## Abstract

**Objectives:**

To explore the value of preoperative magnetic resonance imaging (MRI) characterization of intracranial solitary fibrous tumors (ISFT) and to evaluate the effectiveness of preoperative MRI features in predicting pathological grading.

**Materials and methods:**

This retrospective analysis comprised the clinical and preoperative MRI characterization of 55 patients with ISFT in our hospital, including 27 grade II cases and 28 grade III cases confirmed by postoperative pathology. Variables included age, sex, tumor location, cross-midline status, signal characteristics of T1-weighted imaging (T1WI), T2-weighted imaging (T2WI), T2-fluid-attenuated inversion recovery (T2-FLAIR), and diffusion‑weighted imaging (DWI), peritumoral edema, intralesional hemorrhage, focal necrosis/cystic degeneration, tumor empty vessel, maximum tumor diameter, maximum, minimum, and average values of apparent diffusion coefficient (ADC_max_, ADC_min_, and ADC_mean_), tumors enhancement mode, meningeal tail sign, skull invasion, cerebral parenchymal invasion, and venous sinus involvement. The independent samples *t* test or Mann–Whitney *U* test was performed to compare continuous data between the two groups, and the Pearson chi-squared test or Fisher’s exact test was used to compare categorical data. In addition, bivariate logistic regression was performed to construct a comprehensive model, and receiver operating characteristic (ROC) curves were generated to calculate the areas under the curve (AUCs), thereby determining the value of each parameter in the differential diagnosis of grades II and III ISFT.

**Results:**

The mean age at onset was similar between patients with grades II and III ISFT (46.77 ± 14.66 years and 45.82 ± 12.07 years, respectively). The proportions of men among patients with grades II and III ISFT were slightly higher than those of female patients (male/female: 1.25 [15/12] and 1.33 [16/12], respectively). There were significant differences between grades II and III ISFT in the T2-FLAIR and DWI signal characteristics, maximum, minimum, and average values of the apparent diffusion coefficient (ADC_max_, ADC_min_, and ADC_mean_), tumor location, and skull invasion (*P* = 0.001, *P* = 0.018, *P* = 0.000, *P* = 0.000, *P* = 0.000, *P* = 0.010, and *P* = 0.032, respectively). However, no significant differences were noted between grades II and III ISFT in age, sex, cross-midline status, T1WI and T2WI signal characteristics, peritumoral edema, intralesional hemorrhage, focal necrosis/cystic degeneration, tumor empty vessel shadow, enhancement mode, meningeal tail sign, maximum tumor diameter, brain parenchyma invasion, or venous sinus involvement (all *P* > 0.05). Moreover, binary logistic regression analysis showed that the model accuracy was 89.1% when ADC_min_ was included in the regression equation. Moreover, ROC curve analysis showed that the AUC of ADC_min_ was 0.805 (0.688, 0.922), sensitivity was 74.1%, specificity was 75.0%, and the cutoff value was 672 mm^2^/s.

**Conclusions:**

Grade III ISFT patients displayed more mixed T2-FLAIR signal characteristics and DWI signal characteristics than grade II patients, as shown by higher skull invasion and tumor mass collapse midline distribution and lower ADC_max_, ADC_mean_, and ADC_min_ values. The ADC_min_ value was significant in the preoperative assignment of grades II and III ISFT, thereby contributing to enhanced accuracy in the imaging grading diagnosis of the disease.

## Introduction

Intracranial solitary fibrous tumor (ISFT) is a rare neoplasm originating from the meningeal stroma and comprises a heterogenous group of pleural spindle cell tumors. The latter were first reported in 1931 by Klemperer and Rabin [1] and are derived from dendritic stromal cells expressing the CD34 antigen. Fusion genes of the NGFI-A binding protein-2 signal transduction and transcriptional activator-6 (NAB2-STAT6) were detected early in SFT (solitary fibrous tumor) and hemangiopericytoma (HPC) [2, 3].

In 2016, the World Health Organization (WHO) Central Nervous System Tumor Classification (Fourth Edition) classified ISFT as a type of solid tumor [4]. However, in 2021, the Fifth Edition of this classification (WHO 5th CNS) considered soft tissue pathology, and HPC was removed from the SFT/HPC designation, resulting in an SFT classification only that was divided into WHO grades I ~ III [4]. The grade I ISFT tumor postoperative prognosis was excellent and benign, while grades II and III ISFT tumors were malignant with a relatively poor prognosis. The risk of local recurrence and extracranial metastasis of grade III ISFT was significantly higher than that of grade II ISFT. Furthermore, the progression-free survival and overall survival times of patients with grade III ISFT were considerably shorter than those of grade II ISFT [5, 6]. Therefore, accurately evaluating ISFT classification by preoperative magnetic resonance imaging (MRI) is crucial for treatment planning and optimizing patient prognosis. Few studies have used preoperative MRI characterization to distinguish patients with grades II and III ISFT. In addition, limitations such as small sample size and limited representation in data collection exist [7]。

This study retrospectively analyzed the clinical data and preoperative MRI features of 55 patients with postoperative pathologically proven ISFT in our hospital. The study aimed to investigate the value of preoperative MRI characterization evaluation in predicting grade II–III ISFT classification and identify practical, specific preoperative MRI characteristics. The ultimate aim was to establish an imaging foundation that could inform clinical treatment strategies for ISFT patients.

## Materials and methods

### Study participants

**Fig. 1:**
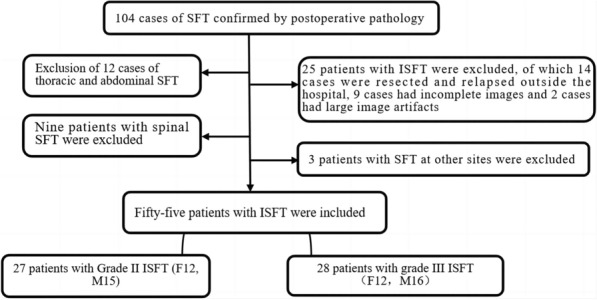
Flow chart for the selection of ISFT patients

This retrospective analysis included 55 patients with ISFT who underwent surgery in our hospital from June 2012 to December 2022. All the patients were pathologically confirmed to have WHO CNS 5th grade II or III and underwent routine and enhanced 3 T MR examinations before surgery. The inclusion and exclusion criteria are shown in Fig. 1. The initial clinical symptoms primarily included headache, epileptic seizures, limb weakness, and cranial nerve dysfunction. This study was approved by the Medical Ethics Committee of the First Affiliated Hospital of Zhengzhou University (approval number: 2019-KY-231) and was exempted from the need to obtain informed consent.

### MRI examination

All patients underwent preoperative superconducting MRI on a 3T MR scanner (MAGNETOM Skyra, Verio, Prisma, Siemens Healthcare, Erlangen, Germany) using a 16-channel cranial coil. The scanning sequence and parameters were as follows: (1) transverse and sagittal T1-weighted imaging (T1WI): slice thickness (ST) = 5 mm, echo time (TE) = 2.5 ms; (2) transverse T2-weighted imaging (T2WI): ST = 5 mm, TE = 2.5 ms; (3) transverse T2-fluid-attenuated inversion recovery (FLAIR): ST = 5 mm, repetition time (TR) = 6500 ms, TE = 85 ms; (4) transverse diffusion-weighted imaging (DWI): ST = 5 mm; TR = 4600 ms, TE = 80 ms, *b* = 0, 1000 s/mm^2^, and apparent diffusion coefficient (ADC) map automatically reconstructed after scanning; and (5) conventional dynamic enhanced (DCE)-MRI: ST = 5 mm, TE = 2.5 ms. Gadolinium meglumine (Gd-DTPA, Guangzhou Kangchen Pharmaceutical Co., China) 0.1 mmol/kg was intravenously injected at a flow rate of 2 ml/s.

### Preoperative MR image analysis

All imaging data were independently evaluated by two associate chief radiologists both with more than 8 years of experience using the picture archiving and communication system (PACS). The following parameters were evaluated: T1WI, T2WI, T2-FLAIR, and DWI signal characteristics with *b* value of 1000 s/mm^2^, tumor location and distribution, midline crossing, peritumoral edema, intralesional hemorrhage, focus necrosis/cystic degeneration, tumor empty vessel, tumor maximum diameter (in millimeters), enhancement mode, and meningeal tail. The maximum apparent diffusion coefficient (ADC_max_), mean ADC (ADC_mean_), and minimum ADC (ADC_min_) were calculated. Tumor location distribution was categorized as supratentorial, subtentorial, or both. Compared to the signal intensity of normal tissues, the signal characteristics were classified as demonstrating hypointensity, isointensity, or hyperintensity. The maximum diameter of the tumor referred to the largest section of the neoplasm, and the ADC value determination was the average value of the solid part of the tumor on DWI with the strongest signal intensity. Brain parenchymal invasion was defined as adherence between the tumor and surrounding brain tissue or invasion of the brain parenchyma. Quantitative values were obtained by averaging the measurements made by the two researchers. With discrepancies in the MRI characterization score, the final assessment was made by agreement between the two researchers.

### Statistical analysis

The statistical analysis was performed using SPSS 26.0 software. The measurement data underwent normality testing with the Shapiro–Wilk *W* test. Normally distributed data were represented by means ± standard deviation (X ± SD), and comparison between the groups was conducted with the independent sample *t* test. Non-normally distributed data were represented by medians (upper and lower quartiles), and the Mann–Whitney *U* test was used for the analysis. In addition, the Fisher exact test was used to compare the classification variables between groups. The parameters with statistically significant differences between groups were incorporated into the variables using the input method, after which a binary logistic regression model was constructed. The variables included in the model were used to plot the subjects’ working characteristic (ROC) curve, and subsequently, the area under the curve (AUC) was obtained to evaluate the diagnostic efficacy of ISFT. The sensitivity and specificity were simultaneously calculated. Last, *P* < 0.05 indicated a statistically significant difference.

## Results

### Clinical characteristics of patients

Among the 55 enrolled ISFT patients, there were more males than females classified as grade II and III (male/female: 1.25 (15/12) in grade II and 1.33 (16/12) in grade III groups); however, there were no significant differences between the sexes (*P* = 0.906) (Table 1). There was no significant difference in the average age of onset between grades II and III ISFT patients (*P* = 0.912), but the median age and age span of the grade II ISFT patients were greater than the grade III ISFT patients (Fig. 2①).

**Table 1:** Clinical characteristics of patients with grade II and grade III ISFT

Variables	G II ISFT	G III ISFT	*χ*^2^/*t* value	*P* value
Sex (F/M)	27 (12/15)	28 (12/16)	0.014	0.906^b^
Age (years)	46.77 ± 14.66	45.82 ± 12.07	0.111	0.912^a^

*G* grade, * ISFT* intracranial solitary fibrous tumor, *F* females, *M* males

^a^Independent sample *t* test

^b^Chi-squared test; *P* value < 0.05 was considered statistically significant

**Fig. 2:**
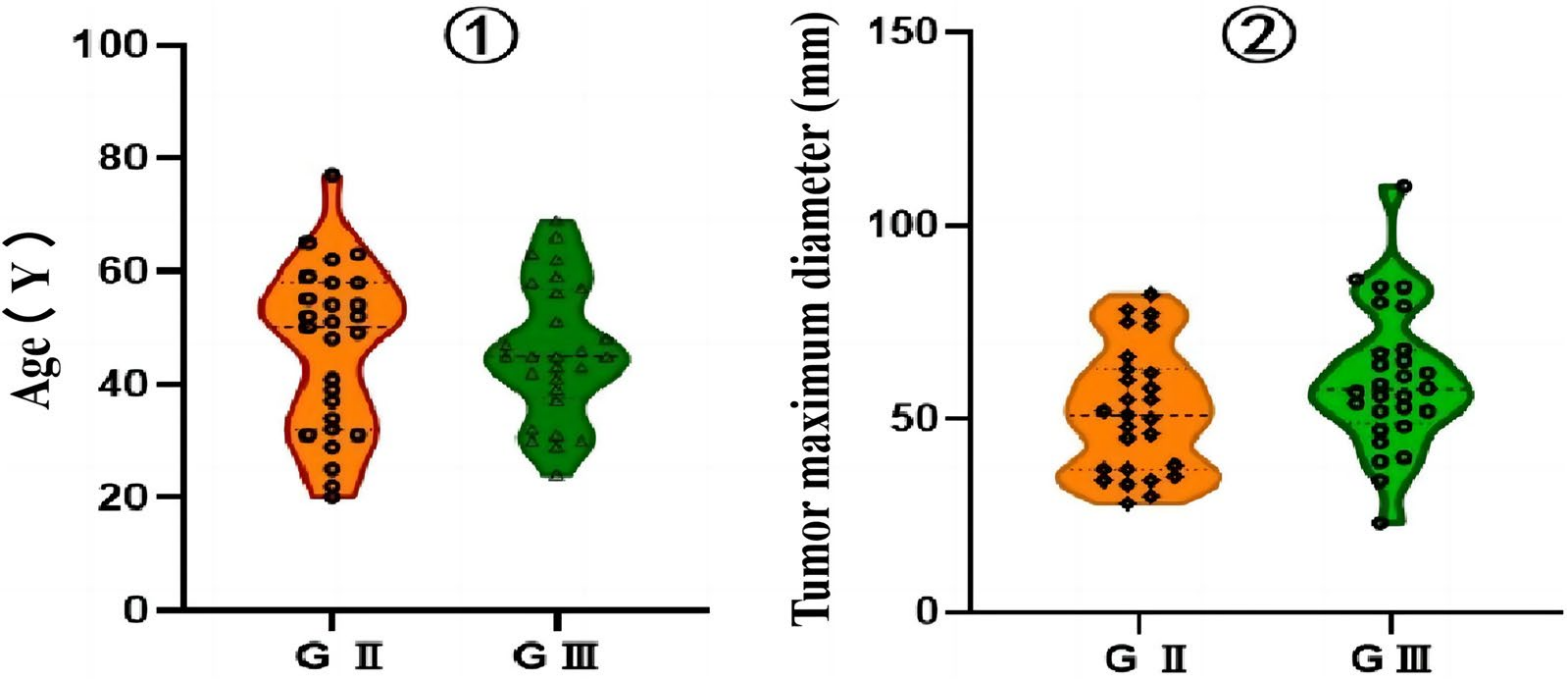
Comparison of ① age (Y) and ② tumor maximum diameter (Y) between patients with Grade II and III ISFT. *Y* year, *G* grade

### Comparison of MR imaging features between grades II and III ISFT patients

**Fig. 3:**
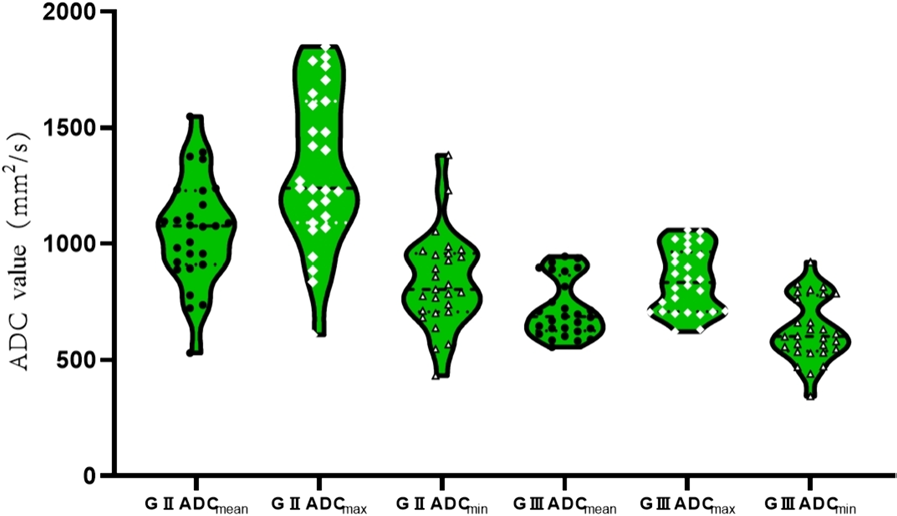
Comparative analysis of ADC_max_, ADC_mean,_ and ADC_min_ values between patients with Grade II and III ISFT. G, grade; ADC_max_, ADC_min_, and ADC_mean_ represents the maximum, minimum, and mean values of the apparent diffusion coefficient (ADC)

The incidence of supratentorial or infratentorial skull invasion in grade III ISFT patients was significantly higher than in grade II ISFT patients (*P* = 0.010, P = 0.032). In addition, there was a significant difference in the T2-FLAIR and DWI signals between grades II and III ISFT patients (*P* = 0.001 and *P* = 0.018, respectively). The ADC_max_, ADC_mean_, and ADC_min_ values in grade III ISFT patients were significantly lower than in grade II ISFT patients (*P* = 0.000, *P* = 0.000, and *P* = 0.000, respectively) (Table 2, Fig. 3).

**Table 2:** Comparison of preoperative MRI parameters and ADC values between Grades II and III ISFT patients

Variables	G II ISFT (*n* = 27)	G III ISFT (*n* = 28)	*χ*^2^/*u*/*t* value	*P* value
Tumor location	1. Supratentorial: 19 2. Infratentorial: 8	1. Supratentorial: 18 2. Infratentorial: 2 3. Supratentorial and infratentorial: 7	9.285	0.010^c^
Cross-midline status	6	12	2.658	0.103^b^
T1WI signal	1. Hypointensity: 4 2. Isointensity: 23, including 16 with heterogeneous intensity	1 Hypointensity: 7 2. Isointensity: 20, including 8 with heterogeneous intensity; 3. Hyperintensity: 1	6.146	0.122^c^
T2WI signal	1. Hypointensity: 3 2. Isointensity: 12, including 9 with heterogeneous intensity; 3. Hyperintensity: 12, including 10 with heterogeneous intensity	1. Hypointensity: 3; 2. Isointensity: 13, including 6 with heterogeneous intensity; 3. Hyperintensity: 12, including 5 with heterogeneous intensity	6.470	0.163^c^
T2-FLAIR signal	1. Hypointensity: 1 2. Isointensity: 15, including 12 with heterogeneous intensity; 3. Hyperintensity: 11, including 8 with heterogeneous intensity	1. Hypointensity: 3 2. Isointensity: 10, including 1 with heterogeneous intensity; 3. Hyperintensity: 15, including 6 with heterogeneous intensity	16.911	0.001^c^
DWI signal (b value = 1000 s/mm^2^)	1. Hypointensity: 8 2. Isointensity: 18 3. Hyperintensity: 1	1. Hypointensity: 1 2. isointensity: 26 3. hyperintensity: 1	7.090	0.018^c^
Peritumoral edema	20	20	0.049	0.826^b^
Intralesional hemorrhage	1	4	/	0.352^c^
Focal necrosis/cystic degeneration	Small cysts: 12 Large cysts: 5	Small cysts: 14 Large cysts: 8	2.675	0.262^b^
Empty vessel	8	11	0.567	0.452^b^
Tumor maximum diameter (mm)	51.96 ± 16.24	60.07 ± 18.22	− 1.747	0.086^a^
ADC_max_ (mm^2^/s)	1322.44 ± 327.78	841.46 ± 136.61	7.149	0.000^a^
ADC_mean_ (mm^2^/s)	1054.04 ± 228.37	721.18 ± 124.38	6.746	0.000^a^
ADC_min_ (mm^2^/s)	836.85 ± 202.29	629.36 ± 136.94	4.469	0.000^a^
Enhancement mode	Inhomogeneous enhancement 27	Inhomogeneous enhancement 28	/	1.000^c^
Meningeal tail sign	2	2	/	1.000^c^
Skull invasion	3	10	4.610	0.032^b^
Cerebral parenchymal invasion	27	27	/	1.000^b^
Venous sinus involvement	6	5	0.164	0.686^b^

*ISFT* intracranial solitary fibrous tumor, T*1WI* T1‑weighted imaging, *T2WI* T2‑weighted imaging, *FLAIR* fluid-attenuated inversion recovery, *DWI* diffusion‑weighted imaging, *G* grade, *ADC*_max_, ADC_min_, and ADC_mean_ represents the maximum, minimum and average values of apparent diffusion coefficient (ADC)

^a^Independent sample *t* test

^b^Chi-squared test

^c^Fisher’s exact test; A *P* value < 0.05 was considered statistically significant

**Fig. 4:**
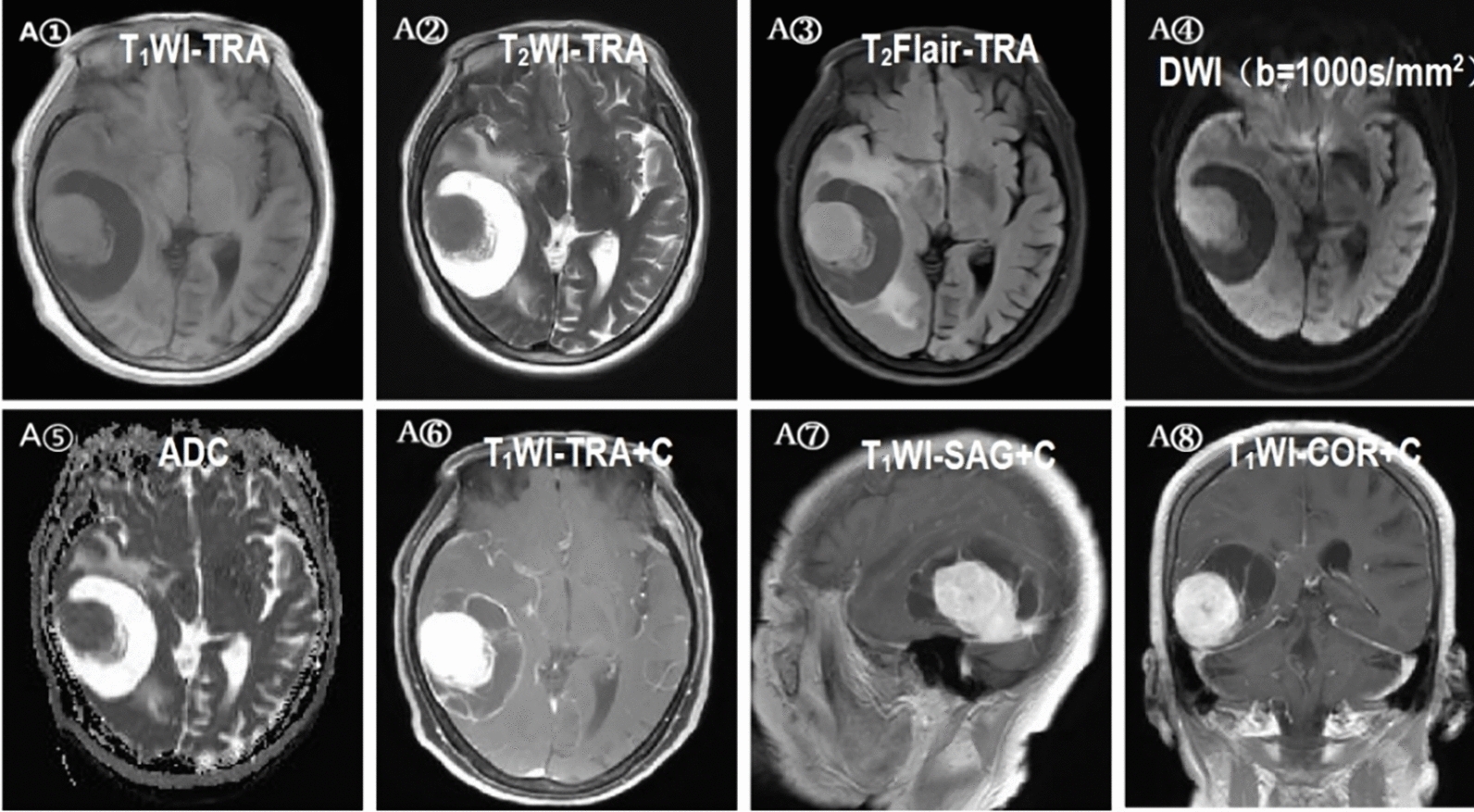
A①–⑧ A 77 year-old woman with GII ISFT. MRI shows a cystic-solid mass with isohomogeneous intensity on the T1WI, T2WI, and T2-FLAIR images (A①–③), slight hyperintensity on the DWI images (*b* value = 1000 s/mm^2^) (A④), slight hypointensity on the ADC maps (A⑤), and slight inhomogeneous enhancement on the contrast-enhanced images (A⑥–⑧). *T1WI* T1‑weighted imaging, *T2WI* T2‑weighted imaging, *FLAIR* fluid-attenuated inversion recovery, *DWI* diffusion‑weighted imaging, *ADC* apparent diffusion coefficient, *TRA* transverse view, *SAG*-sagittal, *COR* coronal

**Fig. 5:**
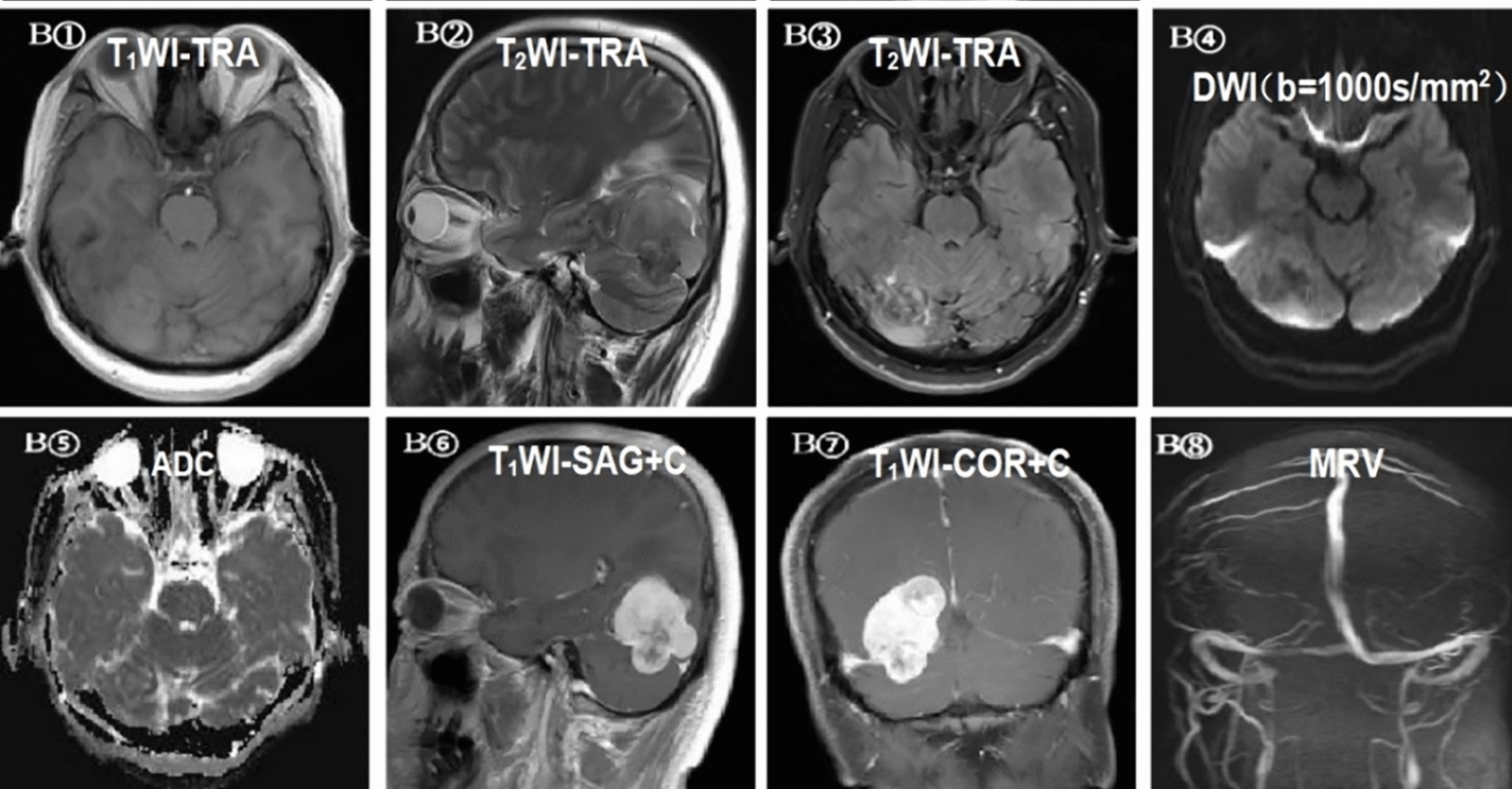
B①–⑧ A 4 3 year-old man with G III ISFT. MRI shows supratentorial and infratentorial solid masses with mixed iso-hypointensity on T1WI and T2WI (B①–②), mixed high- and low-signal intensity on T2-FLAIR (B③), mixed hypointensity on the DWI images (*b* value = 1000 s/mm2) (B④), iso/hyperintensity on the ADC maps (B⑤), and contrast-enhanced MRI (B⑥–⑦) shows obvious inhomogeneous enhancement. (B⑧) MRV images show slight local development in the right transverse sinus. *T1WI, T1* weighted imaging, *T2WI, T2* weighted imaging, *FLAIR* fluid-attenuated inversion recovery, *DWI* diffusion‑weighted imaging, *ADC* apparent diffusion coefficient, *MRV* magnetic resonance venography (*TRA* transverse, *SAG*, sagittal, *COR* coronal)

There were no significant differences between grades II and III ISFT patients in the cross-midline condition, T1 signal characteristics, T2 signal characteristics, peritumoral edema, intralesional hemorrhage, focal necrosis/cystic degeneration, tumor emptying vascular shadow, enhancement mode, meningeal tail sign, maximum tumor diameter, brain parenchyma invasion, and venous sinus involvement (Table 2, Figs. 4, 5). Notably, the average maximum tumor diameter of grade III ISFT patients was larger than that of grade II ISFT patients (Fig. 2②).

### Binary logistic regression model to evaluate the effectiveness of preoperative MRI characterization in predicting grades II and III ISFT patients

**Fig. 6:**
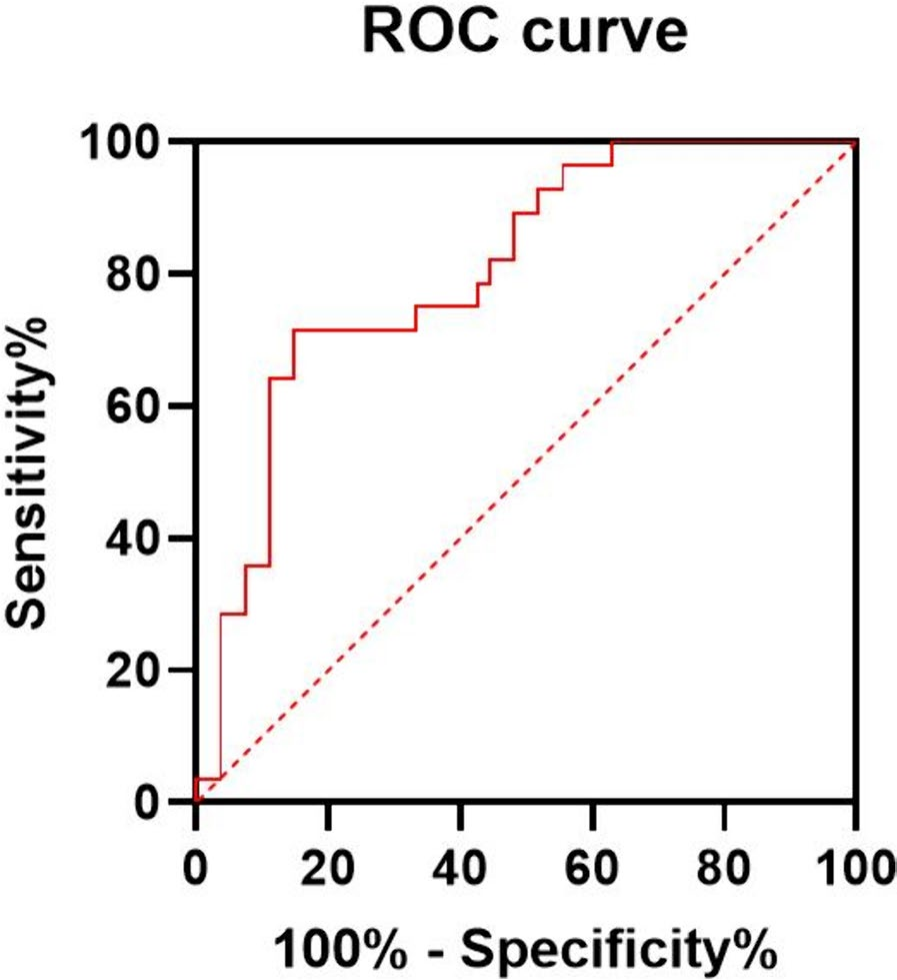
Receiver operating characteristic (ROC) curve of ADC_min_ in discriminating patients with Grades II and III ISFT

The prediction model was developed using binary logistic regression analysis, considering tumor location, skull invasion, T2-FLAIR signal characteristics, DWI signal characteristics, and ADC_max_, ADC_mean_, and ADC_min_ values. The Variance Inflation Factor (VIF) values were 1.268, 1.311, 1.228, 1.577, 16.336, 32.033, and 8.261, respectively. Variables with VIF < 10 were selected for binary logistic regression analysis. The reference category for multicategory variables was the last such classification and the final ADC_min_ value as the single best quantitative parameter was chosen to enter the equation. The model’s prediction accuracy for grades II and III ISFT patients was 89.1% (Table 3). The ROC curve exhibited an AUC of 0.805 (0.688, 0.922) for the ADC_min_ value, with a sensitivity of 85.2%, and a specificity of 71.4%. The cutoff value for ADC_min_ was determined to be 672 mm^2^/s. (Fig. 6).

**Table 3:** Analysis of variables in hierarchical binary logistic regression equation for Grades II and III ISFT

Variables	OR value	OR value (95% CI)	*Wald*	*P* value
ADC_min_ (mm^2^/s)	0.990	0.982 ~ 0.998	6.417	0.011
T2-FLAIR signal	/*	/	7.856	0.097
Tumor location	/*	/	1.206	0.547
Skull invasion	0.078	0.002 ~ 3.995	1.616	0.204
DWI signal (b value = 1000 s/mm^2^)	/*	/	0.037	0.982

*DWI* diffusion‑weighted imaging, *FLAIR* fluid-attenuated inversion recovery, ADC_min_, minimum values of apparent diffusion coefficient. /* represents categorical variables > 3 groups; *OR* odds ratio, *CI* confidence intervals

A *P* value < 0.05 was considered statistically significant

## Discussion

Solitary fibrous tumor (SFT) has been reported in almost every anatomical site in the body, with the pleura being the most common, accounting for approximately 30% of cases. Other commonly involved sites include the dura (27%), abdominal cavity (20%), trunk (10%), limbs (8%), and the head and neck (5%) [8]. ISFT accounts for less than 1% of all intracranial tumors, and surgical excision is the primary treatment for this neoplasm [4, 9]. Significant differences in tumor biological behavior, prognosis, postoperative survival rate, and tumor progression exist between grades II and III ISFT patients [7, 10]. Therefore, practical preoperative evaluation of ISFT grades can provide an essential theoretical basis for patients to develop personalized treatment plans.

In this study, clinical information, and the preoperative MRI characterization of 55 patients with ISFT grades II and III were retrospectively analyzed. There were statistically significant differences in tumor location distribution, skull invasion, T2-FLAIR signal characteristics, DWI signal characteristics, and ADC_max_, ADC_mean_, and ADC_min_ values between the two groups. Additionally, binary logistic regression analysis was performed based on classifying ISFT cases into grades II and III, revealing that the ADC_min_ value had a higher diagnostic efficacy for this categorization.

### Comparison of general clinical and preoperative MRI features of grades II and III ISFT patients

This study revealed that the onset age of ISFT patients ranged from 20 to 77 years old, with an average age of 46.02 years. The male prevalence was slightly higher than that of females (male/female: 1.24). Still, the median age and age span of grade II ISFT patients were larger than those of grade III patients, different from the findings of Liu et al. [11] but consistent with the results of Thway K et al. [12]. This gender distribution was consistent with a recent population-based study [13].

ISFT patients exhibit various clinical manifestations, primarily due to tissue compression or increased intracranial pressure caused by tumor growth. Among the symptoms observed, headache is the most common. In contrast, other clinical symptoms, such as dizziness, visual impairment, epilepsy, limb weakness, and hearing impairment, are related to nerve invasion caused by the tumor. In addition, previous studies reported several unusual symptoms, including language disorder, olfactory loss, memory loss, hyponatremia, and hypoglycemia [14], which were not seen in this study.

The preoperative MRI findings of ISFT patients in this study revealed a distribution of tumors encompassing the skull base, parasagittal sinus, venous sinus, cerebral falx, ventricle, and cerebellopontine angle. A total of 67% of these tumors were identified as supratentorial and 18% infratentorial. Notably, grade III ISFT patients exclusively manifested supratentorial and infratentorial tumors. Among them, 43% of grade III ISFT patients displayed midline-crossing tumors, a significantly higher proportion than the 22% observed in grade II ISFT patients, different from the findings reported by Zhang et al. [15]. This difference might be due to a substantial disparity in the number of grade II ISFT patients between this study and others.

ISFT grows slowly, often resulting in a larger tumor diameter [16]. In this study, ISFT tumor sizes ranged between 23 to 110 mm, with an average diameter of 56 mm, and the average diameter in grade III ISFT patients marginally exceeded that in grade II patients. Consistent with previous studies, ISFT predominantly displayed low signals in T1WI and mixed signals in T2WI and T2-FLAIR [17]. The ‘‘black-and-white sign’’ in T2-weighted imaging remains a specific diagnostic indicator for ISFT [18]. This study found that high signal intensity in the T2-FLAIR signal was greater in grade III ISFT patients.

Past studies have demonstrated an association between high signal intensity in T2-weighted images with increased collagen content in fibrous tissue, intrafocal hemorrhage, cystic degeneration or relatively fresh fibrosis, and the distribution of high cells within the ‘‘antler’’ capillary area [19, 20]. In this study, DWI using a high *b* value (b = 1000 s/mm^2^) revealed predominantly iso-signal characteristics in ISFT patients. The main factors affecting water molecule diffusion in tissue included the nucleus-to-cytoplasm ratio and macromolecular substance content in the cytoplasm. However, diffusion was susceptible to the T_2_ permeation effect, necessitating more accurate use of the ADC value [21].

This study found significantly lower tumor ADC_max_, ADC_mean_, and ADC_min_ values in patients with grade III versus grade II ISFT, with statistically significant differences. Yamashita et al. [22] reported that the nuclear/cytoplasmic ratio and tumor cell density of tumor cells were significantly correlated with the ADC values. Notably, grade III ISFT exhibited a higher nuclear/cytoplasmic ratio and increased tumor cell density, restricting extracellular and intracellular water molecule diffusion, resulting in lower ADC values than grade II ISFT, consistent with observations made by Zhang et al. [10].

In this study, 35% of grades II and III ISFT patients demonstrated inhomogeneous enhancement, which might have been related to increased vascularity within the tumor [23]. Notably, conspicuous peritumoral edema was observed in grades II and III ISFT patients, likely due to obstruction of the adjacent venous sinus and the elevated expression of vascular endothelial growth factor. In addition, cystic degeneration and necrosis were common in patients with grades II and III ISFT, with a higher incidence of cystic degeneration in grade III ISFT patients, consistent with findings from Zhou et al. [24]. In addition, the rate of macrocystic degeneration in grade III ISFT patients was higher than in grade II ISFT patients. Potential contributing factors to this discrepancy included accelerated tumor growth, immature vascular erosion, and thrombosis events, resulting in tumor cell ischemia and hypoxia, with a possible correlation between malignancy degree of the tumor and the prominence of this phenomenon. Intrafocal hemorrhage was infrequent in the study, consistent with previous studies, and this finding was potentially linked to perivascular fibrosis evident on pathology [8, 25].

ISFT presentations commonly featured uneven enhancement, while the meningeal tail sign caused by long-term chronic meningeal stimulation was rare. The imaging manifestations included meningeal and cranial invasion, accounting for 23.6% (13/55) of cases. The skull invasion rate was markedly higher in grade III patients, at 35.7% (10/28) compared to 11.1% (3/27) in grade II patients. In addition, approximately 20.0% (11/55) of tumors invaded the venous sinus due to the tumor’s rich blood supply and varied cell growth rates along its periphery. The tumor’s local growth characteristics can lead to invasive changes in the adjacent meninges and bones, consistent with invasive skull destruction in about 44.4% (4/9) of ISFT patients, as reported by Clarençon et al. [26].

### Efficacy of the binary logistic regression model in predicting grades II and III ISFT

A binary logistic regression analysis integrating clinical information and preoperative MRI features was conducted to predict pathological grades II and III ISFT. Among the variables considered, ADC_min_ emerged as the sole factor incorporated into the binary logistic regression equation. This model demonstrated an accuracy rate of 89.1% in predicting the pathological grades of patients with grades II and III ISFT. In addition, the diagnostic efficiency of ADC_min_ for distinguishing between pathological grades II and III ISFT, as evaluated by ROC curve analysis, was found to be 0.805, sensitivity was 85.2%, specificity was 71.4%, and the cutoff value was 672 mm^2^/s.

The ADC values reflect the diffusion capacity of water molecules within tumor cells. Grade III ISFT tumor cells display a denser irregular arrangement, a higher nucleo -plasma ratio, greater nuclear heteromorphic appearance, and increased cellular proliferation compared to grade II SFT tumor cells. These factors contribute to restricted water molecule diffusion within tumors [15]. Moreover, findings by Chen et al. [27] and Higano et al. [28] suggested that the ADC_min_ corresponds to tumor peak cell density, exhibiting a negative correlation with the cell proliferation index and reflecting the tumor differentiation degree. Therefore, the ADC_min_ can serve as an effective tool in predicting pathological grades II and III ISFT.

Variables such as tumor location, skull invasion, T2-FLAIR signal characteristics, and DWI signal characteristics do not predict pathological grades II and III ISFT. This lack of distinction could be attributed to how grades II and III ISFT can infiltrate the brain parenchyma and peripheral perforator vessels, resulting in subtle changes in preoperative MRI signals insufficient for grade differentiation. In addition, grades II and III ISFT can exhibit invasive changes, resulting in bone destruction.

Although this study found that grade III ISFT was more aggressive than grade II, this finding was insufficient to effectively distinguish the two grades. In summary, ADC_min_ emerged as a straightforward and efficient preoperative diagnostic tool for predicting pathological grades II and III ISFT. This variable served as a basis for the patients’ imaging prognosis and subsequent diagnosis and treatment planning.

## Limitations

This study had several limitations. It was a retrospective, single-center study with a limited sample size. There may have been limitations in extracting quantitative indicators related to preoperative MRI tumor features. Future prospective, multi-center studies involving larger sample sizes and refined quantitative methodologies might help improve preoperative MRI case classification diagnostic accuracy.

## Conclusions

This study analyzed the general clinical characteristics and preoperative MRI findings of grades II and III ISFT patients while developing a binary logistics regression model to identify effective predictors for pathological grading based on postoperative pathology. Variations in tumor location distribution, skull invasion, T2-FLAIR signal characteristics, DWI signal characteristics, and ADC_max_, ADC_mean_, and ADC_min_ values were observed between grades II and III ISFT patients. Notable among these indicators, ADC_min_ emerged as the sole effective predictor for image-based pathological grading. The ADC_min_ was highly efficient in predicting grades II and III tumors in ISFT patients. This finding holds substantial importance in facilitating preoperative imaging grading of ISFT patients, assisting in precise diagnosis and treatment plans, and evaluating prognosis outcomes, offering valuable imaging indicators for practical evaluation.

## Data Availability

All data were acquired through the PACS system, and patient information was redacted.

## References

[1] Klemperer P, Coleman BR. Primary neoplasms of the pleura a report of five cases. Am J Ind Med. 1992;22(1):1–31. 10.1002/ajim.4700220103. 1415270 10.1002/ajim.4700220103

[2] Stout AP, Murray MR. Hemangiopericytoma: a vascular tumor featuring zimmermann’s pericytes. Ann Surg. 1942;116(1):26–33. 10.1097/00000658-194207000-00004. 17858068 10.1097/00000658-194207000-00004 PMC1543753

[3] Robinson DR, Wu YM, Kalyana-Sundaram S, et al. Identification of recurrent NAB2-STAT6 gene fusions in solitary fibrous tumor by integrative sequencing. Nat Genet. 2013;45(2):180–5. 10.1038/ng.2509. 23313952 10.1038/ng.2509 PMC3654808

[4] Louis DN, Perry A, Wesseling P, et al. The 2021 WHO Classification of Tumors of the Central Nervous System: a summary. Neuro Oncol. 2021;23(8):1231–51. 10.1093/neuonc/noab106. 34185076 10.1093/neuonc/noab106 PMC8328013

[5] Giordan E, Marton E, Wennberg AM, et al. A review of solitary fibrous tumor/hemangiopericytoma tumor and a comparison of risk factors for recurrence, metastases, and death among patients with spinal and intracranial tumors. Neurosurg Rev. 2021;44(3):1299–312. 10.1007/s10143-020-01335-x. 32556679 10.1007/s10143-020-01335-x

[6] Sung KS, Moon JH, Kim EH, et al. Solitary fibrous tumor, hemangiopericytoma: treatment results based on the WHO classification. J Neurosurg. 2016. 10.3171/2017.9.JNS171057. 27834597 10.3171/2017.9.JNS171057

[7] Shin DW, Kim JH, Chong S, et al. Intracranial solitary fibrous tumor/hemangiopericytoma: tumor reclassification and assessment of treatment outcome via the 2016 WHO classification. J Neurooncol. 2021;154(2):171–8. 10.1007/s11060-021-03733-7. 34417710 10.1007/s11060-021-03733-7

[8] Ronchi A, Cozzolino I, Zito Marino F, et al. Extrapleural solitary fibrous tumor a distinct entity from pleural solitary fibrous tumor an update on clinical, molecular and diagnostic features. Ann Diagn Pathol. 2018. 10.1016/j.anndiagpath.2018.01.004. 29660566 10.1016/j.anndiagpath.2018.01.004

[9] Ratneswaren T, Hogg FRA, Gallagher MJ, et al. Surveillance for metastatic hemangiopericytoma-solitary fibrous tumors-systematic literature review on incidence, predictors and diagnosis of extra-cranial disease. J Neurooncol. 2018;138(3):447–67. 10.1007/s11060-018-2836-2. 29551003 10.1007/s11060-018-2836-2

[10] Zhang B, Li S, Zhang P, et al. Preoperative MRI semantic features in predicting postoperative tumor progression of intracranial grade II-III solitary fibrous tumors/hemangiopericytoma. Chin J Med Imaging. 2021;29(07):651–8. 10.3969/j.issn.1005-5185.2021.07.002. 10.3969/j.issn.1005-5185.2021.07.002

[11] Liu J, Wu S, Zhao K, Wang J, Shu K, Lei T. Clinical features, management, and prognostic factors of intracranial solitary fibrous tumor. Front Oncol. 2022;30(12): 915273. 10.3389/fonc.2022.915273. 10.3389/fonc.2022.915273 PMC9197442 35712477

[12] Thway K, Ng W, Noujaim J, et al. The current status of solitary fibrous tumor: diagnostic features, variants, and genetics. Int J Surg Pathol. 2016;24(4):281–92. 10.1177/1066896915627485. 26811389 10.1177/1066896915627485

[13] Kinslow CJ, Bruce SS, Rae AI, Sheth SA, McKhann GM, Sisti MB, Bruce JN, Sonabend AM, Wang TJC. Solitary-fibrous tumor/hemangiopericytoma of the central nervous system: a population-based study. J Neurooncol. 2018;138(1):173–82. 10.1007/s11060-018-2787-7. 29427152 10.1007/s11060-018-2787-7

[14] Gopakumar S, Srinivasan VM, Hadley CC, Anand A, Daou M, Karas PJ, Mandel J, Gopinath SP, Patel AJ. Intracranial solitary fibrous tumor of the skull base: 2 cases and systematic review of the literature. World Neurosurg. 2021;149:e345–59. 10.1016/j.wneu.2021.02.026. 33609763 10.1016/j.wneu.2021.02.026

[15] Zhang J, Liu H, Zhou Z. The value of MinADC combined with conventional MRI in predicting of intracranial SFT/HPC grading. J Clin Radiol. 2020. 10.13437/j.cnki.jcr.2020.07.005. 10.13437/j.cnki.jcr.2020.07.005

[16] Ge W, Yu DC, Chen G, et al. Clinical analysis of 47 cases of solitary fibrous tumor. Oncol Lett. 2016;12(4):2475–80. 10.3892/ol.2016.4967. 27698815 10.3892/ol.2016.4967 PMC5038456

[17] Li X, Tan L, Ouyang X, Jiang J, et al. Magnetic resonance features of meningeal solitary fibrous tumors. Oncol Lett. 2018;15(6):8825–32. 10.3892/ol.2018.8426. 29805622 10.3892/ol.2018.8426 PMC5950533

[18] Weon YC, Kim EY, Kim HJ, Byun HS, Park K, Kim JH. Intracranial solitary fibrous tumors: imaging findings in 6 consecutive patients. AJNR Am J Neuroradiol. 2007;28(8):1466–9. 10.3174/ajnr.A0609. 17846192 10.3174/ajnr.A0609 PMC8134371

[19] Tariq MU, Din NU, Abdul-Ghafar J, et al. The many faces of solitary fibrous tumor; diversity of histological features, differential diagnosis and role of molecular studies and surrogate markers in avoiding misdiagnosis and predicting the behavior. Diagn Pathol. 2021;16(1):32. 10.1186/s13000-021-01095-2. 33879215 10.1186/s13000-021-01095-2 PMC8059036

[20] Nawashiro H, Nagakawa S, Osada H, et al. Solitary fibrous tumor of the meninges in the posterior cranial fossa: magnetic resonance imaging and histological correlation—case report. Neurol Med Chir. 2000;40(8):432–4. 10.2176/nmc.40.432. 10.2176/nmc.40.432 10979268

[21] Lee EJ, TerBrugge K, Mikulis D, et al. Diagnostic value of peritumoral minimum apparent diffusion coefficient for differentiation of glioblastoma multiforme from solitary metastatic lesions. AJR Am J Roentgenol. 2011;196(1):71–6. 10.2214/AJR.10.4752. 21178049 10.2214/AJR.10.4752

[22] Yamashita Y, Kumabe T, Higano S, Watanabe M, Tominaga T. Minimum apparent diffusion coefficient is significantly correlated with cellularity in medulloblastomas. Neurol Res. 2009;31(9):940–6. 10.1179/174313209X382520. 19138469 10.1179/174313209X382520

[23] Liu Y, Tao X, Shi H, et al. MRI findings of solitary fibrous tumours in the head and neck region. Dentomaxillofac Radiol. 2014;43(3):20130415. 10.1259/dmfr.20130415. 24720608 10.1259/dmfr.20130415 PMC4064629

[24] Zhou JL, Liu JL, Zhang J, et al. Thirty-nine cases of intracranial hemangiopericytoma and anaplastic hemangiopericytoma: a retrospective review of MRI features and pathological findings. Eur J Radiol. 2012;81(11):3504–10. 10.1016/j.ejrad.2012.04.034. 22658867 10.1016/j.ejrad.2012.04.034

[25] Machado I, Nieto-Morales G, Cruz J, et al. Controversial issues in soft tissue solitary fibrous tumors: a pathological and molecular review. Pathol Int. 2020;70(3):129–39. 10.1111/pin.12894. 31904167 10.1111/pin.12894

[26] Clarençon F, Bonneville F, Rousseau A, et al. Intracranial solitary fibrous tumor: imaging findings. Eur J Radiol. 2011;80(2):387–94. 10.1016/j.ejrad.2010.02.016. 20303226 10.1016/j.ejrad.2010.02.016

[27] Chen Z, Ma L, Lou X, et al. Diagnostic value of minimum apparent diffusion coefficient values in prediction of neuroepithelial tumor grading. J Magn Reson Imaging. 2010;31(6):1331–8. 10.1002/jmri.22175. 20512884 10.1002/jmri.22175

[28] Higano S, Yun X, Kumabe T, et al. Malignant astrocytic tumors: clinical importance of apparent diffusion coefficient in prediction of grade and prognosis. Radiology. 2006;241(3):839–46. 10.1148/radiol.2413051276. 17032910 10.1148/radiol.2413051276

